# Amazon Basin climate under global warming: the role of the sea surface temperature

**DOI:** 10.1098/rstb.2007.0037

**Published:** 2008-02-11

**Authors:** Phil P Harris, Chris Huntingford, Peter M Cox

**Affiliations:** 1 Centre for Ecology and HydrologyWallingford, Oxon OX10 8BB, UK; 2 School of Engineering, Computer Science and Mathematics, University of ExeterHarrison Building, North Park Road, Exeter EX4 4QF, UK

**Keywords:** climate change, tropical forest, sea surface temperature, carbon cycle, global warming

## Abstract

The Hadley Centre coupled climate–carbon cycle model (HadCM3LC) predicts loss of the Amazon rainforest in response to future anthropogenic greenhouse gas emissions. In this study, the atmospheric component of HadCM3LC is used to assess the role of simulated changes in mid-twenty-first century sea surface temperature (SST) in Amazon Basin climate change.

When the full HadCM3LC SST anomalies (SSTAs) are used, the atmosphere model reproduces the Amazon Basin climate change exhibited by HadCM3LC, including much of the reduction in Amazon Basin rainfall. This rainfall change is shown to be the combined effect of SSTAs in both the tropical Atlantic and the Pacific, with roughly equal contributions from each basin. The greatest rainfall reduction occurs from May to October, outside of the mature South American monsoon (SAM) season. This dry season response is the combined effect of a more rapid warming of the tropical North Atlantic relative to the south, and warm SSTAs in the tropical east Pacific. Conversely, a weak enhancement of mature SAM season rainfall in response to Atlantic SST change is suppressed by the atmospheric response to Pacific SST. This net wet season response is sufficient to prevent dry season soil moisture deficits from being recharged through the SAM season, leading to a perennial soil moisture reduction and an associated 30% reduction in annual Amazon Basin net primary productivity (NPP). A further 23% NPP reduction occurs in response to a 3.5°C warmer air temperature associated with a global mean SST warming.

## 1. Introduction

First-generation coupled climate–carbon cycle models indicate that carbon cycle feedbacks may accelerate anthropogenic climate change through the twenty-first century (Friedlingstein *et al.* 2006). The principal feedbacks are from the terrestrial biosphere, and one model in particular (Hadley Centre coupled climate–carbon cycle model, HadCM3LC, Cox *et al.* 2000) simulates an almost complete loss of forest from the Amazon Basin between 2050 and 2100 under conditions of locally reduced precipitation and increased air temperature. While future drying of the Amazon Basin is not common to all general circulation models (GCMs) contributing to the Intergovernmental Panel on Climate Change Fourth Assessment Report (IPCC AR4; Christensen *et al.* 2007), it remains useful to study the processes that contribute to the strong Amazon climate change exhibited by Cox *et al*. (2000, 2004)

Betts *et al.* (2004) estimated that global carbon cycle feedbacks and vegetation structural changes in HadCM3LC contribute to approximately 40% of the simulated Amazon Basin precipitation reduction. This indicates that the majority of the regional drying is part of a wider atmosphere–ocean response to greenhouse warming. The HadCM3LC trend in the twenty-first century Pacific sea surface temperature (SST) has been described as ‘El Niño like’ (Collins 2005), and Cox *et al.* (2004) showed that a future weakening of Amazon Basin rainfall during December to February (DJF) is correlated with a progressive weakening of the equatorial Pacific east–west SST gradient. They suggest that Amazon Basin drying is related to this SST change through Walker circulation perturbations, similar to those thought to relate observed South American rainfall and El Niño-Southern Oscillation (Aceituno 1998; Grimm 2003).

However, the observed seasonal cycle and interannual variation in Amazon Basin precipitation are linked additionally to tropical Atlantic SST (e.g. Nobre & Shukla 1996; Marengo *et al.* 2001). Most recently, it was suggested that prolonged dry periods in parts of the Amazon Basin through 2005 were related to an anomalously warm tropical north Atlantic (Marengo 2006). The potential contribution of Pacific and Atlantic SST change to Amazon drying in HadCM3LC is noted by Li *et al.* (2006) in an analysis of AR4 model output. However, such an analysis of transient GCM simulations is unable to distinguish the relative roles of concurrent SST changes in each ocean basin.

To investigate the roles of future Pacific and Atlantic SST change in Amazon Basin drying, we run the atmospheric component of HadCM3LC with a range of SST boundary conditions derived from the transient simulations of Cox *et al.* (2000). The model and methods used are described in §2, and the HadAM3 control climate is described in §3. The results of the HadAM3 simulations with different SST boundary conditions are described in §4, and conclusions are given in §5.

## 2. Model and methods

The atmospheric GCM used in this study is HadAM3 (Pope *et al.* 2000) including the MOSES2 land surface scheme (Essery *et al.* 2001). HadAM3 is a hydrostatic, primitive equation model with discretization of 19 vertical levels and 96×73 horizontal gridboxes, corresponding to a gridbox size of 3.75° longitude×2.5° latitude.

In a control simulation (CONT), HadAM3 is integrated for 10 model years using the GISST 1961–1990 climatology (Parker *et al.* 1995, v. 2.3b) as the SST boundary condition. Unless stated otherwise, atmospheric CO_2_ concentration in each of these simulations was set to the HadCM3LC 1961–1990 mean value of 348 ppmv. The initial 3 years of the simulation are excluded from the analysis to allow the model to ‘spin up’. A further six experimental integrations are made by adding to the GISST climatology SST anomalies (SSTAs) based on the climatologies of HadCM3LC-simulated SST change between 1961–1990 and 2040–2059. This future period, centred on 2050, is chosen to simulate an Amazon Basin climate change sufficient to initiate forest loss in HadCM3LC, but also one through which the effects of vegetation change on Amazon rainfall are weak (see Betts *et al.* 2004).

**Figure 1:**
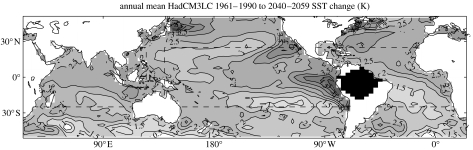
Change in annual mean HadCM3LC-simulated SST for 2040–2059 relative to 1961–1990.Contours are plotted every 0.5°C. The monthly mean SSTAs applied in the HadAM3 experiments MWLA, MW, LA, ATL, PAC and IND are based on this change, as described in table 1. Shaded land pixels indicate the region used for calculating Amazon Basin area means, and dashed lines indicate regions of each basin where SSTAs were applied in ATL, PAC and IND.

The annual mean HadCM3LC SST change, shown in figure 1, exhibits warming in almost all ocean gridboxes corresponding to a global annual mean warming of 1.7°C. Notable regional features in the tropics are an east–west gradient in Pacific warming, a north–south gradient in Atlantic warming, and a near-uniform warming of the Indian Ocean. The maximum eastward extent of the equatorial Pacific warming occurs through the Northern Hemisphere winter (DJF), the same season as the peak SSTAs in this region observed during El Niño events. Tropical Atlantic SST changes enhance the existing SST seasonal cycle and produce a northward shift in the location of the maximum SST throughout the year.

The study assesses the contribution of these SST features to modelled Amazon climate change using the SST boundary conditions summarized in table 1. A full HadCM3LC SST change simulation (MWLA) is an atmosphere-only reproduction of the HadCM3LC response to mean 2040–2059 SST change. An additional simulation is made using MWLA SSTAs but with atmospheric CO_2_ concentration increased to the HadCM3LC 2040–2059 mean value of 592 ppmv to verify that HadAM3 reproduces the full HadCM3LC Amazon Basin climate change. The full MWLA SST change is separated further into two components: a global mean warming (MW) and gridbox local anomalies to this global mean (LA). In three further simulations, the local anomalies are restricted to the tropical basins of the Atlantic (ATL), Pacific (PAC) and Indian (IND) Oceans between 25° S and 25° N, and bounded by 15° latitude and longitude linear relaxations to zero anomaly for preventing unrealistic spatial discontinuities in SST.

## 3. Simulated control climate

A detailed global analysis of the HadAM3 control climate (as reproduced by the CONT simulation herein) is given by Pope *et al.* (2000), and only the model biases over South America and the tropical Atlantic are described in this section. Amazon Basin area means quoted in this section and following ones are calculated over the model gridboxes indicated in figure 1. The observation datasets used herein are the CMAP observation-only climatology for precipitation (Xie & Arkin 1997) and the CRU land-only climatology for 1.5 m air temperature (*T*_a_ from v. CRU-CL-1.0; New *et al.* 1999).

The CONT simulation produces tropical precipitation with annual mean wet bias of 1.0 mm d^−1^ over tropical land. Over South America, the annual mean precipitation rate north of 40° S is 4.2 mm d^−1^ corresponding to a 0.1 mm d^−1^ wet bias. This is compared with a dry bias of 0.4 mm d^−1^ for this region in HadCM3LC. Over the Amazon Basin, defined here by the shaded gridboxes in figure 1, the CONT annual mean precipitation rate of 5.3 mm d^−1^ has a dry bias of 0.1 mm d^−1^, which is weaker than the dry bias of 0.8 mm d^−1^ in HadCM3LC. Simulated peak mature South American monsoon (SAM) precipitation rates of 9–10 mm d^−1^ from DJF agree with CMAP rates in magnitude, but are located approximately 5° too far south. This produces a ‘dipole’ of dry–wet biases from the Amazon to 20° S for both CONT and HadCM3LC. When estimated biases in CMAP annual mean rainfall of 10–20% over Amazon Basin are considered (Xie & Arkin 1997), both CONT and HadCM3LC are acceptable simulations of SAM precipitation in timing and magnitude.

Annual mean *T*_a_ over the Amazon Basin is 25.3°C in CONT, corresponding to a cool bias of 0.4°C compared with the CRU dataset, and which contrasts with a HadCM3LC warm bias of 0.8°C. This cool bias over the whole basin comprises strong cold biases of 2°C in the Andes and weaker warm biases of <1°C in the Amazon lowlands. New *et al.* (1999) noted that the interpolation method used to construct the CRU dataset tends to underestimate the air temperature lapse rate, resulting in air temperatures that are probably too warm at high altitudes. Accounting for this bias would tend to reduce the cool bias in the Amazon Basin mean. In the Amazon lowlands, monthly mean *T*_a_ values are within 1°C of CRU data throughout the year.

## 4. Results

### (a) Rainfall response to global SST changes

An annual Amazon Basin rainfall reduction (table 2) of 1.4 mm d^−1^ (−30%) in HadCM3LC is reproduced by HadAM3 as an MWLA drying of 1.1 mm d^−1^ (−21%). The spatial extent of this drying is coincident in the two models, and covers the whole Amazon Basin. The additional simulation (not shown) with atmospheric CO_2_ increased to 592 ppmv produces a drying of 1.4 mm d^−1^ (−27%), indicating that much of the additional drying in HadCM3LC is due to the effects of elevated CO_2_ in the coupled model. This is consistent with the results of Betts *et al.* (2004) who estimated that the stomatal response to increased CO_2_ enhances the drying by approximately 20%.

**Figure 2:**
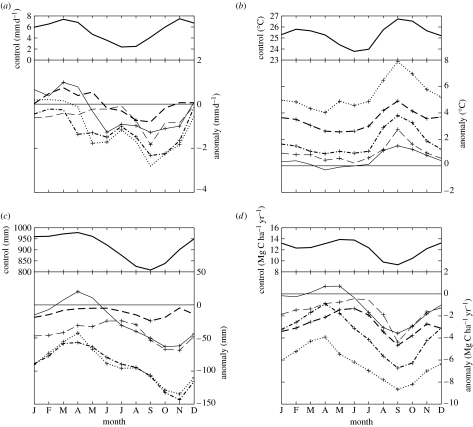
Simulated monthly mean Amazon Basin (*a*) rainfall rate (mm d^−1^) (*b*) 1.5 m air temperature (°C), (*c*) total soil moisture content (mm) and (*d*) NPP (Mg C ha^−1^ yr^−1^). Upper panels show CONT climatologies and lower panels show anomalies in each experiment relative to CONT. Thick lines indicate simulations using global SSTAs and thin lines indicate simulations with SSTAs restricted to a tropical basin. Crosses indicate anomalies significant at the 99% level using Student's *t*-test. Thick line, CONT; dotted line, MWLA; thick dashed line, MW; dash dotted line, LA; thin line, ATL; thin dashed line, PAC.

Monthly mean rainfall anomalies for MWLA in figure 2*a* show that the greatest drying of up to 2.8 mm d^−1^ occurs between May and November, outside of the peak months of the SAM, and through which observed interannual variance in Amazon Basin rainfall is the greatest. This dry season response has been noted previously for HadCM3LC by Li *et al.* (2006). Weak rainfall increases through the mature SAM months of January to March reflect enhanced SAM rainfall with no shift in location of the peak from the southern edge of the Amazon Basin (as defined in figure 1). By contrast, HadCM3LC exhibits a 1.1 mm d^−1^ drying through these months (not shown), probably related to a stomatal response to increased CO_2_. Rainfall reductions through May and June correspond to an early recession of the SAM.

Comparison with the MW and LA simulations shows that these May to November rainfall reductions are part of an atmosphere response to regional patterns of SST warming, rather than the increase in global mean SST. Moreover, this corresponds to a delayed onset of the Amazon Basin monsoon and a longer dry season. The effect of global mean SST increase in MW is to enhance the existing seasonal cycle of rainfall over the Amazon Basin. However, these monthly changes are not statistically significant and the effect on the annual mean Amazon Basin rainfall is relatively small.

### (b) Response to tropical SST changes

Annual mean rainfall rates in table 2 show Amazon Basin drying in ATL and PAC of 0.3 and 0.6 mm d^−1^, respectively. Neither Pacific nor Atlantic SST gradient changes alone explain the strength of rainfall reduction in response to global SST gradient changes. Moreover, the sum of ATL and PAC anomalies explains only 80% of drying in LA, indicating that the response to global SSTAs is not simply the response to Atlantic and Pacific SSTAs summed through all seasons. These annual mean rainfall responses suggest a stronger response to Pacific SST gradients changes than Atlantic gradient changes; however, it is shown in §4*b*(i),(ii) that there are significant differences between seasons.

An IND annual mean drying of 0.2 mm d^−1^ corresponds to a weakening of DJF rainfall over northeast Brazil rather than the Amazon Basin. This is consistent with a modelled rainfall response to warm Indian Ocean SSTAs following El Niño events (Spencer *et al.* 2004). However, because there is no statistically significant response over the Amazon Basin, the IND simulation is not considered further here.

#### (i) Amazon Basin dry season

Monthly mean Amazon Basin rainfall anomalies for ATL in figure 2*a* show a consistent drying of approximately 1 mm d^−1^ from June to November, and a similar 1 mm d^−1^ drying in PAC from August to November. Both correspond to a longer dry season, which is a strong determinant of vegetation type in the tropics (Sternberg 2001), but the ATL dry season has a more severe annual minimum than that in PAC.

**Figure 3:**
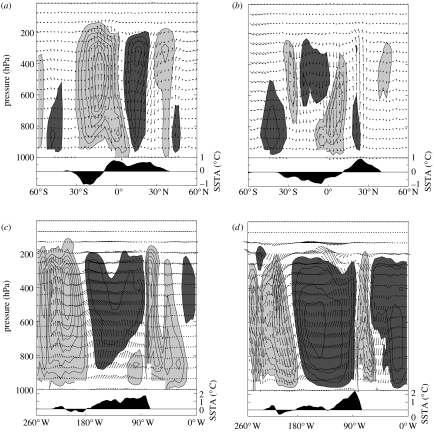
(Upper panels) Zonal mean atmospheric Hadley circulation (ATL) and anomalies from 70° W to 45° W for (*a*) SAM months of January to March and (*b*) dry season months of July to September. Contours show CONT vertical velocity, plotted every 1 Pa s^−1^; light and dark shadings indicate ascent and descent rates, respectively, of >1 Pa s^−1^. Arrows show ATL–CONT anomalies in meridional wind–vertical velocity fields. Shaded region shows 65° W to 20° W zonal mean SSTA from ATL using right-hand scale. (Lower panels) Meridional mean atmospheric Walker circulation (PAC) and anomalies from 5° S to 5° N for (*c*) SAM months of January to March and (*d*) dry season months of July to September.

Associated with these dry season rainfall decreases in the ATL and PAC simulations are changes in the Hadley and Walker circulations, respectively (figure 3). In response to Atlantic SSTAs over the tropical South America/Atlantic region, there is an overall strengthening of the Hadley circulation and associated rainfall increases over ocean. The effect of this over South America is indicated in figure 3*b* by increased ascent from 10° to 30° N over warm SSTAs and increased descent between 30° S and 5° N across the same latitudes as cool SSTAs. There are also stronger 950 hPa cross-equatorial southerlies and a shift in intertropical convergence zone (ITCZ) rainfall from South America north of the equator towards the Caribbean. These changes could affect Amazon rainfall through both increased upper troposphere convergence and reduced lower troposphere moisture convergence (Hastenrath 2000).

Observed Atlantic ITCZ rainfall, unlike the mean change in ATL, exhibits little interannual variation in latitude through the dry season (Chiang *et al.* 2002). From July to September, a strong meridional gradient in tropical SST supports ITCZ convection over only a narrow band of latitudes. As a result, the peak SST and the associated ITCZ rainfall are moved only by relatively large SSTAs. For the future change, ATL SSTAs (figure 3*b*, lower panel) are sufficient to shift northwards the maximum SST from 7.5° to 12° N and strengthen the inter-hemispheric SST gradient, resulting in the significant drying over northern South America. The ATL inter-hemispheric gradient also persists for one to two months longer at the end of the dry season, which leads to a delay in the SAM development phase (Li & Fu 2004).

The atmospheric response to Pacific SSTAs through the dry season (figure 3*d*) exhibits increased ascent over the warm equatorial east Pacific SSTAs from 135° to 85° W and descent over cool west Pacific SSTAs. These anomalies correspond to a weaker, but extant, Pacific Walker circulation. However, the strongest east Pacific ascent increase from July to September occurs over the warmest SSTs approximately 10° N, away from the equatorial SST ‘cold tongue’. This results in a removal of the 600–200 hPa mean ascent over equatorial South America and the associated rainfall reduction in figure 2*a*. Because the east Pacific convection anomaly is located north of the Equator, the Andes form less of a barrier to lower troposphere connections with the equatorial and northern Amazon than for warm SSTA in the equatorial east Pacific (Fu *et al.* 2001). It is probable that this helps limit significant PAC Amazon Basin rainfall reductions to the dry season.

#### (ii) South American monsoon season

Through the mature SAM season (JFM), there are few significant changes in Amazon Basin rainfall (figure 2*a*) from any SSTAs, with the exception of ATL for March. This March rainfall increase due to Atlantic SSTAs comprises a 1 mm d^−1^ increase in SAM maximum rainfall on the southern edge of the basin (not shown), with an associated strengthening of the existing Hadley circulation (figure 3*a*). Chiang *et al.* (2002) noted that a broad Atlantic SST maximum about the equator during these months supports ITCZ convection over a range of latitudes, resulting in the greatest observed interannual variation in ITCZ location from January to April. Warm equatorial SSTAs (figure 3*a*, lower panel) enhance the climatological equatorial maximum SST and increase the SST gradient away from the equator favouring an enhanced monsoon. However, the relatively short simulation lengths make it difficult to distinguish a significant mean wet season change from internal variability through these months, despite relatively strong ATL circulation anomalies.

### (c) Amazon Basin surface response to SST change

Sections 4*a*,*b* describe how mid-twenty-first century SST change in HadCM3LC is associated with a 21% reduction in annual mean Amazon Basin rainfall. However, rainfall change initiates modelled forest loss only indirectly through the effect of soil moisture limitation on vegetation net primary productivity (NPP). Table 2 shows that the MWLA rainfall reduction coincides with a 10% reduction in soil moisture but a proportionately greater 52% reduction in basin mean NPP. Comparison with simulations MW and LA indicates that this NPP change comprises approximately a 30% reduction from SST gradients and a 23% reduction from global mean warming.

Amazon Basin monthly mean air temperature (figure 2*b*) exhibits an MW warming of 3–5°C through the year with no significant soil moisture reduction (figure 2*c*). While MWLA soil moisture reductions are attributable to SST gradient changes in all months, the corresponding NPP reductions in LA are 2.5–3.0 Mg C ha^−1^ yr^−1^ weaker than those in MWLA. This difference may be accounted for by the NPP response to mean SST warming. This indicates that there would be a significantly reduced Amazon Basin NPP in HadCM3LC, even in the absence of SST gradient changes in the Atlantic and Pacific. However, the NPP reduction required to initiate forest loss would occur later in the twenty-first century. It is interesting to note that a simulation with increased atmospheric CO_2_ (not shown) exhibited a weaker NPP reduction of 33% due to the effect of CO_2_ fertilization.

The rainfall responses to tropical Atlantic and Pacific SSTAs produce relatively weak annual mean NPP reductions (table 2) of 9 and 13%, respectively. Each of these reductions is unlikely to be sufficient to cause forest loss. These NPP reductions are primarily in response to reduced dry season soil moisture (figure 2*c*), although there is some PAC wet season drying restricted to land around the Amazon River mouth. The drier ATL soil through the dry season is offset by the wet season rainfall increase to produce a relatively weak (3%) annual mean reduction. These contrast markedly with the strong, perennial LA soil moisture reduction produced when the combined ATL and PAC dry season responses are propagated from year to year. This indicates that the contribution of SST-related rainfall change to HadCM3LC Amazon Basin forest loss requires both the Atlantic and Pacific SST gradient changes examined herein.

## 5. Conclusions

A scenario as striking as the predicted loss of Amazon forest by Cox *et al.* (2000), along with the associated social and economic implications for the region, warrants further investigation. This is particularly important because regional climate projections for tropical South America are not consistent across different GCMs contributing to the IPCC AR4.

This study used atmospheric GCM simulations to assess the effect of simulated future SST change on Amazon Basin climate. Particular attention was given to the roles of SST gradient change in the tropical Atlantic and Pacific. We conclude that several concurrent SST conditions are sufficient to reduce Amazon Basin NPP to a level at which rainforest is unsustainable. These conditions include strengthening of inter-hemispheric SST gradients in the Atlantic and east Pacific through the Amazon Basin dry season and global mean SST warming. The establishment of these Atlantic and Pacific SSTAs under greenhouse warming is uncertain; current coupled atmosphere–ocean GCMs disagree on the signs of SST gradient change across both the zonal equatorial Pacific (Collins 2005) and the meridional Atlantic (Good *et al.* 2008). This study highlights the importance of reducing uncertainty in the prediction of future SST change in order to understand and quantify the risk of climate change-induced loss of Amazon rainforest.

## Figures and Tables

**Table 1:** Description of SST boundary conditions used in the HadAM3 experiments. (‘MW’ and ‘LA’ indicate global mean warming and local anomalies respectively.)

experiment	SST boundary condition
CONT	GISST
MWLA	GISST+full SST change
MW	GISST+global mean warming
LA	GISST+full change−global mean
ATL	as LA, but ΔSST in tropical Atlantic only
PAC	as LA, but ΔSST in tropical Pacific only
IND	as LA, but ΔSST in tropical Indian Ocean only

**Table 2:** Simulated Amazon Basin (see figure 1 for region definition) precipitation rate, air temperature, total soil moisture content (SMC) and NPP from each experiment. (Differences from control values are shown in parentheses. HadCM3LC anomalies are taken from the transient simulation for 2040–2059 relative to 1961–1990.)

experiment	rainfall (mm d^−1^)	1.5 m T (°C)	top 3 m SMC (mm)	NPP (Mg C ha^−1^ yr^−1^)
CONT	5.34	25.3	912.8	12.2
HadCM3LC	3.25 (−1.39)	31.1 (+4.7)	791.0 (−78.0)	5.1 (−4.4)
MWLA	4.23 (−1.11)	30.7 (+5.4)	822.1 (−90.7)	5.8 (−6.3)
MW	5.35 (+0.01)	28.8 (+3.5)	900.8 (−12.0)	9.4 (−2.8)
LA	4.18 (−1.16)	27.1 (+1.8)	820.5 (−92.3)	8.6 (−3.6)
ATL	5.01 (−0.33)	25.8 (+0.5)	889.0 (−23.8)	11.1 (−1.1)
PAC	4.76 (−0.58)	26.3 (+1.0)	870.0 (−42.8)	10.6 (−1.6)
IND	5.16 (−0.18)	25.5 (+0.2)	903.4 (−9.4)	11.9 (−0.3)
